# Impact of selected non-steroidal anti-inflammatory pharmaceuticals on microbial community assembly and activity in sequencing batch reactors

**DOI:** 10.1371/journal.pone.0179236

**Published:** 2017-06-22

**Authors:** Cong Jiang, Jinju Geng, Haidong Hu, Haijun Ma, Xingsheng Gao, Hongqiang Ren

**Affiliations:** State Key Laboratory of Pollution Control and Resource Reuse, School of the Environment, Nanjing University, Jiangsu, PR of China; Purdue University, UNITED STATES

## Abstract

This study covers three widely detected non-steroidal anti-inflammatory pharmaceuticals (NSAIDs), diclofenac (DCF), ibuprofen (IBP) and naproxen (NPX), as NSAIDs pollutants. The objective is to evaluate the impact of NSAIDs at their environmental concentrations on microbial community assembly and activity. The exposure experiments were conducted under three conditions (5 μg L^-1^ DCF, 5 μg L^-1^ DCF+5 μg L^-1^ IBP and 5 μg L^-1^ DCF+5 μg L^-1^ IBP+ 5 μg L^-1^ NPX) in sequencing batch reactors (SBRs) for 130 days. Removals of COD and NH_4_^+^-N were not affected but total nitrogen (TN) removal decreased. IBP and NPX had the high removal efficiencies (79.96% to 85.64%), whereas DCF was more persistent (57.24% to 64.12%). In addition, the decreased removals of TN remained the same under the three conditions (*p* > 0.05). The results of oxidizing enzyme activities, live cell percentages and extracellular polymeric substances (EPS) indicated that NSAIDs damaged the cell walls or microorganisms and the mixtures of the three NSAIDs increased the toxicity. The increased Shannon-Wiener diversity index suggested that bacterial diversity was increased with the addition of selected NSAIDs. Bacterial ribosomal RNA small subunit (16S) gene sequencing results indicated that Actinobacteria and Bacteroidetes were enriched, while *Micropruina* and *Nakamurella* decreased with the addition of NSAIDs. The enrichment of Actinobacteria and Bacteroidetes indicated that both of them might have the ability to degrade NSAIDs and thereby could adapt well with the presence of NSAIDs.

## Introduction

The non-steroidal anti-inflammatory drugs (NSAIDs) are the most commonly used drugs worldwide. Among them, diclofenac (DCF), ibuprofen (IBP) and naproxen (NPX) are frequently detected in the environment as trace emerging contaminants [1]. DCF has been in the watch list of compounds in EU and had already harmfully affected several environmental species at concentration of < 1 μg L^-1^ [2]. IBP usually has the highest influent concentrations among pharmaceuticals [3]. NPX is an acid pharmaceutical like DCF and IBP. It is often detected in the environment with DCF and IBP [4]. The growing production and application of these three NSAIDs raises the risk of discharging them into wastewater treatment plants (WWTPs). WWTPs are not only the gathering points of these NSAIDs but also the main source of the NSAIDs to receiving water bodies [5]. In the last few decades, concern is growing over the determination and occurrence of NSAIDs in the environment and in WWTPs. In accordance with observations, the NSAIDs are usually detected at levels from ng L^-1^ to μg L^-1^ in WWTPs [6–8]. It was also found that these three NSAIDs have the tendency to accumulate in aquatic bodies, leading to an increase in the possibility of human exposures [9,10].

The fate of NSAIDs in WWTPs would be affected by various parameters (e.g., temperature, pH, biomass concentration, biodegradability, cation-exchange properties et al.). During wastewater treatment processes, NSAIDs would be transformed by abiotic processes (e.g., sorption, isomerization/epimerization, hydrolytic) and biotic transformation/degradation [11]. Tixier et al. [10] found that DCF in water was eliminated mainly by photo-transformation and the removal of NPX might be biodegradation. Anaerobic soils exhibited high biological degradation of IBP and the half-life was 41.2 days, while a half-life of 121.9 days was determined under aerobic conditions [12]. Domaradzka et al. [13] found that biotransformation/biodegradation of polycyclic NSAIDs was caused by fungal microorganisms, mainly white-rot fungi. Their biotransformation pathways most often include hydroxylation catalyzed by P-450 monooxygenases and hitherto information about the bacterial transformation/biodegradation of polycyclic NSAIDs is limited [13].

Most pharmaceuticals are released into WWTPs without specific treatment and WWTPs are not designed to remove pharmaceuticals. Currently, the effect of pharmaceuticals (such as ofloxacin, norfloxacin, ciprofloxacin, tetracycline, sulfamethoxazole et al.) on WWTP performance or the efficiency of plans for their removal has been extensively researched [8, 14, 15]. Arriaga et al. [16] reported a decrease of chemical oxygen demand (COD) removal in conventional activated sludge (CAS) reactors when treating wastewater with pharmaceuticals than without pharmaceuticals. Zhang et al. [15] investigated the effect of antibiotics on reactor performance and found that COD and NH_4_^+^-N removals were not affected by 5 μg L^-1^ of antibiotics but decreased significantly with the presence of higher concentration antibiotics (10 mg L^-1^). However, few researches have addressed the impact of NSAIDs on reactor performances.

In most instances, municipal WWTPs use biological wastewater treatment processes to remove nitrogen, phosphorus and organic pollutants where microbial components of activated sludge drive the key processes. The existence and activity of microorganisms that can biodegrade NSAIDs play the most important role in the fate of NSAIDs in wastewater than their inherent physic-chemical properties and environmental factors [17]. Lawrence et al. [18] investigated the impact of DCF (10 and 100 μg L^-1^) on biofilm communities and found that 100 μg L^-1^ DCF had significant effects on bacterial community compared to the control. Meanwhile, the use of carbon sources was significantly depressed (*p* < 0.05) with the presence of 100 μg L^-1^ DCF. Therefore, it is reasonable to infer that the shifts in microbial community might be detrimental to WWTP performance, such as COD removal rate.

The bacterial population is made up of macromolecules extracellular polymeric substances (EPS). Delgado et al. [19] reported that the production of EPS content was a reaction to environmental stress. Oxidative stress response could be also used to indicate low toxicity to moderate toxicity of selected pharmaceuticals (especially DCF) by increasing activities of oxidative stress enzymes [20]. But a shortage of research exists on the impacts of NSAIDs on oxidative stress enzymes in activated sludge. Zhao et al. [21] used membrane bioreactors seeded with aerobic granular sludge to treat wastewater containing five kinds of pharmaceuticals and personal care products (PPCPs) and the result indicated that the presence of PPCPs causes structural changes in microorganisms. Wang and Gunsch [22] found that mixtures of four pharmaceutically active compounds (PhACs) would inhibit nitrification and affect the ammonia-oxidizing bacteria (AOB) community structure in sequencing batch reactors (SBRs) but did not research the impact of PhACs on the non-nitrifying microbial community. To date, very few studies have focused on the effect of these NSAIDs on the microbial community structure of conventional activated sludge. In fact, further investigation into microbial activity, microbial communities and activated sludge system responses to pharmaceuticals is crucial.

This study operated lab-scale SBRs for 130 d to culture activated sludge with selected NSAIDs at environmental concentration. COD, NH_4_^+^-N and TN were measured regularly during operation time. The microbial activity and community in activated sludge were observed at steady state. The objectives of this study were to: (1) learn more information about the overall reactor performance with additions of selected NSAIDs, (2) investigate changes of microbial activity in sludge reflected by extracellular polymeric substances (EPS) and oxidizing enzyme activities, and (3) determine the shifts and diversity of the microbial community in activated sludge to selected NSAIDs. The present investigation contributes to the further understanding of the emerging contamination effect on the wastewater treatment process.

## Materials and methods

### Chemicals and consumables

DCF, IBP and NPX (all above 98% pure) were obtained from Sigma Aldrich (Steinheim Germany). Methanol, acetic acid and acetone (all HPLC grade) were purchased from Merck (Darmstadt, Germany). Milli-Q water produced from a Millipore purification system (Billerica, CA, USA) was used throughout except for synthetic wastewater. Other chemicals (analytical grade) were supplied by Nanjing Chemical Reagent Factory, China. Oasis hydrophilic-lipophilic balance (HLB) solid phase extraction (SPE) cartridges (200 mg, 6 mL) were obtained from Waters (Milford, MA, USA). Individual analyte stock solutions (100 mg L^-1^) were prepared in methanol and stored under refrigeration at 4°C.

### Set-up and operation of sequencing batch reactors (SBRs)

Exposure experiments were operated in four lab-scale SBRs with a working volume of 4 L for 130 d. The SBRs were conducted at room temperature (20±5°C) and at a hydraulic retention time of 10 h. The solid retention time (SRT) was set to 20 d by a daily manual purge. The reactors were continuously conducted with a 12 h cycle containing 30 min feeding, 600 min aeration, 60 min settling and 30 min decanting. The initial pH was controlled at 7.5±0.5 by using NaHCO_3_. DO concentration was maintained at 3.5±0.5 mg L^-1^ by controlling air flow rates.

The reactors were inoculated with seed sludge collected from the aeration tank of a municipal WWTP in Nanjing, China. The mixed liquor suspended solids (MLSS) levels were 3.5±0.2 g L^-1^. The initial concentration of COD, NH_4_^+^–N and total phosphorus (TP) in synthetic wastewater were 300±12, 20±1 and 3.5±0.5 mg L^-1^, respectively. One liter of synthetic wastewater also contained 12 mg of CaCl_2_·2H_2_O, 35 mg of MgSO_4_·7H_2_O and 0.3 mL of a trace nutrient solution. The trace nutrient solution was described by [23]. Selected NSAIDs (DCF, IBP and NPX) were added with 2 mL of 10 mg L^-1^ stock solutions which were diluted by 4 L of synthetic wastewater and finally maintained with the influent pharmaceutical concentration of 5 μg L^-1^. R_0_ was the control without pharmaceuticals and R_1_–R_3_ were set in different mixtures of selected NSAIDs added into the synthetic wastewater during the feeding period (Table 1). After operating for three SRT cycles, the reactors could be defined as stable [15].

**Table 1:** Pharmaceutical concentrations and mixture in SBRs.

Reactor code	Pharmaceutical concentrations and mixture
R_0_	Control
R_1_	5 μg L^-1^ DCF
R_2_	5 μg L^-1^ DCF+5 μg L^-1^ IBP
R_3_	5 μg L^-1^ DCF+5 μg L^-1^ IBP +5 μg L^-1^ NPX

R_0_ operated without NSAIDs, R_1_ operated at DCF of 5 μg L^-1^ in the reactor influent, R_2_ operated at 5 μg L^-1^ DCF and 5 μg L^-1^ IBP in the reactor influent, R_3_ operated at the 5 μg L^-1^ mixtures of DCF, IBP and NPX in the reactor influent.

### Determination of oxidizing enzyme activities in activated sludge

All enzymes were extracted from activated sludge during the stable stage in triplicate. The activities of superoxide dismutase (SOD) and succinate dehydrogenase (SDH) were evaluated by corresponding detection kits obtained from the Nanjing Bioengineering Institute (China). The catalog numbers for SOD and SDH are A001-3 and A022, respectively. All enzyme activities were normalized by total protein concentration. This study used the Micro BCA Protein Assay Kit obtained from Nanjing Bioengineering Institute to the distribution of proteins (PN) (China).

### EPS content and live/dead cell distributions on activated sludge

Extracellular polymeric substances (EPS) are extracted from activated sludge [24]. In most instances, EPS content is presented by exopolysaccharide (PS) and their proteins (PN) [25]. The phenol-sulfuric method with glucose as the standard was used to detect PS content (glucose equivalent) [26]. A modified Lowry method using bovine serum albumin as a standard was used to measure PN content [27].

Live and dead cell distributions in activated sludge after pharmaceuticals exposure was investigated with epifluorescence microscopy. This study used the LIVE/DEAD BacLight kit (Invitrogen Molecular Probes, USA), which consists of propidium iodide (PI) and SYTO9. Green fluorescing SYTO9 was used for assessing the live cells, whereas red fluorescing PI was used to assess the dead cells. Twenty random fields were chosen and observed, and the rate of live/dead cells in activated sludge was calculated by quantifying the area of each image with the image processing software Image J (National Institutes of Health, America). The final result of live/dead cells was assessed by the Green area/(Green area + Red area).

### Analytical methods

COD, NH_4_^+^–N, TN and MLSS were measured by Standard Methods for the Examination of Water and Wastewater [28]. pH and temperature values were detected with pH meters (FE20, METTLER TOLEDO Inc., USA).

For evaluating the concentration of DCF, IBP and NPX, influent and effluent samples were collected from each reactor and filtered through a 0.22 um pore-size filter (Xinya, Shanghai, China). Before determining the NSAIDs concentrations, the pH of each sample was adjusted to 2 with 2 M HCl. Pharmaceuticals were pretreated by solid phase extraction (SPE) and the detailed method was carried out according to [5]. The final extraction samples were transferred to the 2 mL amber vial and stored at 4°C until analysis. The samples of influent and effluent were directly injected into the Ultra Performance Liquid Chromatography-Mass Spectrometry system (Waters Corp., Milford, MA, USA). This analysis method has been described by [29] and is still a current practice.

The results were recorded as an average ± standard deviation (SD). One-way analysis of variance (ANOVA) was used to assess the homogeneity of variance with significance level of 5% (*p* < 0.05). Statistical analyses were performed using SPSS statistics 22.0.

### Procedure of high-throughput 16S rRNA gene amplicon sequencing

Activated sludge samples were collected from each reactor at the stable period in triplicate for microbial analysis. Processes contained DNA extractions, 16 S rRNA gene PCR amplifications and PCR product purifications. The total genomic DNA was extracted by the Fast DNA^™^ Spin Kit for Soil (MP Biomedicals, Santa Ana, CA, USA). A NanoDrop 2000 UV-Vis spectrophotometry (Thermo Scientific, Wilmington, DE, USA) was used to determine the concentration and quality of DNA. The DNA samples were stored at –20°C until analysis. The V1-V2 hypervariable region of the 16S rRNA gene was used with primers and different 8-base barcodes and a Guanine were linked to the 5′ end of each primer. The information of primers was as follows: forward primer (5′-AGAGTTTGATYMTGGCTCAG-3′) and reverse primer (5′-TGCTGCCTCCCGTAGGAGT-3′) [25]. The purified products were used in the Illumina high-throughput sequencing (Miseq, Illumina Inc., USA) at Jiangsu Zhongyijinda Analytical and Testing Limited Company (Jiangsu, China). Illumina high-throughput sequencing used Miseq V2 Reagent Kit (500 cycles). Sequence length is 250 bp paired-end reads. Sequencing data was estimated using Sickle to remove the bases of low quality (Q<25) and any sequences with more than one N (https://github.com/najoshi/sickle). Mothur program was used for sequence demultiplexing and filtration. Lastly, sequence data used in this study has been submitted to Sequence Read Archive (SRA) with the accession number PRJNA 383079. The Shannon-Wiener index (H) was used to indicate the microbial species diversity of activated sludge [25].

## Results and discussion

### Reactor performance

Due to the microbial adaptation to the reactor condition, removal of COD, NH_4_^+^-N and TN fluctuated during the first 60 days. After that, the removal of COD, NH_4_^+^–N and TN tended to be stable. In most instances, the operation of reactors would achieve a stable stage after an adaptation time of at least two SRT in the biological process [30]. The average removals are illustrated in Table 2.

**Table 2:** Average removal of COD, NH_4_^+^–N and TN during stable stage of each reactor.

Samples	COD removal (%)	NH_4_^+^-N removal (%)	TN removal (%)
R_0_	91.18±1.53	99.82±0.39	64.04±4.21
R_1_	92.88±1.31	99.93±0.08	55.12±3.39*
R_2_	92.74±1.27	99.30±0.15	53.19±4.11*
R_3_	92.41±1.78	99.18±0.77	53.88±4.27*

The reactor operation achieved a stable stage after 60 days. The results of COD, NH4^+^–N and TN removals were recorded as an average ± SD. The average and SD values were calculated from 60^th^ day to 130^th^ day (the stable stage).

* statistically different from the control (*p* <0.05)

As shown in Table 2, COD and NH_4_^+^–N removals were all over 90% in each sample (*p* > 0.05), but the removals of TN decreased from 64.04% to 53.19% (*p* < 0.05) and there was no obvious difference in R_1_, R_2_ and R_3_ of TN removal (*p* > 0.05). The results indicated that the selected NSAIDs of environmental concentration had little effect on COD and NH_4_^+^–N removals, but decreased TN removal. It seems that TN removal more easily affected than COD removal. A possible explanation of unchanged COD removal is that the sum of heterotrophs responsible for organic matter removal were not affected by the addition of selected NSAIDs of environmental concentration, and the decreased TN removal might be due to the decrease of denitrifying microorganisms. Similarly, Zhang et al. [15] used SBRs to evaluate the impact of tetracycline and sulfamethoxazole on bioreactor effluent quality and found that COD and NH_4_^+^–N removals appeared unchanged (*p* > 0.05) with addition of 5 and 50 μg L^-1^ tetracycline and sulfamethoxazole.

The selected NSAID removals in the stable stage are gathered in Table 3. It is evident that the removals of DCF, IBP and NPX were from 57.27% to 64.12%, 85.64% to 88.12% and 79.96%, respectively. For DCF and IBP, the removals slightly decreased with the mixtures of selected NSAIDs but the removal changes were not significant.

**Table 3:** Average removals of selected NSAIDs in the stable stage of each reactor.

		R_0_	R_1_	R_2_	R_3_
Influent (μg L^-1^)	DCF	-	5.02±0.08	5.06±0.11	5.03±0.14
	IBP	-	-	4.97±0.06	5.01±0.17
	NPX	-	-	-	5.09±0.03
Effluent (μg L^-1^)	DCF	-	1.80±0.10	2.06±0.11	2.15±0.10
	IBP	-	-	0.59±0.04	0.72±0.07
	NPX	-	-	-	1.02±0.04
Removal efficiencies (%)	DCF	-	64.12±2.58	59.22±3.09	57.27±0.77
	IBP	-	-	88.12±0.70	85.64±1.01
	NPX	-	-	-	79.96±0.37

The reactor operation achieved a stable stage after 60 days. The results of NSAIDs removals were recorded as an average ± SD.

The elimination of selected NSAIDs was contributed to various removal mechanisms including sorption, biodegradation and abiotic degradation. DCF, IBP and NPX contain carboxyl groups belonging to acidic functional groups, which could decrease the surface pH to increase their absorption coefficient [31]. Many studies investigated the degradation of NSAIDs and the results indicated that DCF was much more difficult to remove [16, 32, 33]. Kimura et al. [32] found that IBP had higher removal rate than DCF in conventional activated sludge. Elimination of IBP was mainly by biodegradation and elimination of DCF relied on sorption but not biodegradation. Similarly, Kruglova et al. [33] detected high biodegradation for IBP and no biodegradation for DCF and carbamazepine in nitrifying activated sludge under 12°C temperature conditions.

### Microbial activity

#### Effect of selected NSAIDs on oxidizing enzyme activities in activated sludge

**Fig 1:**
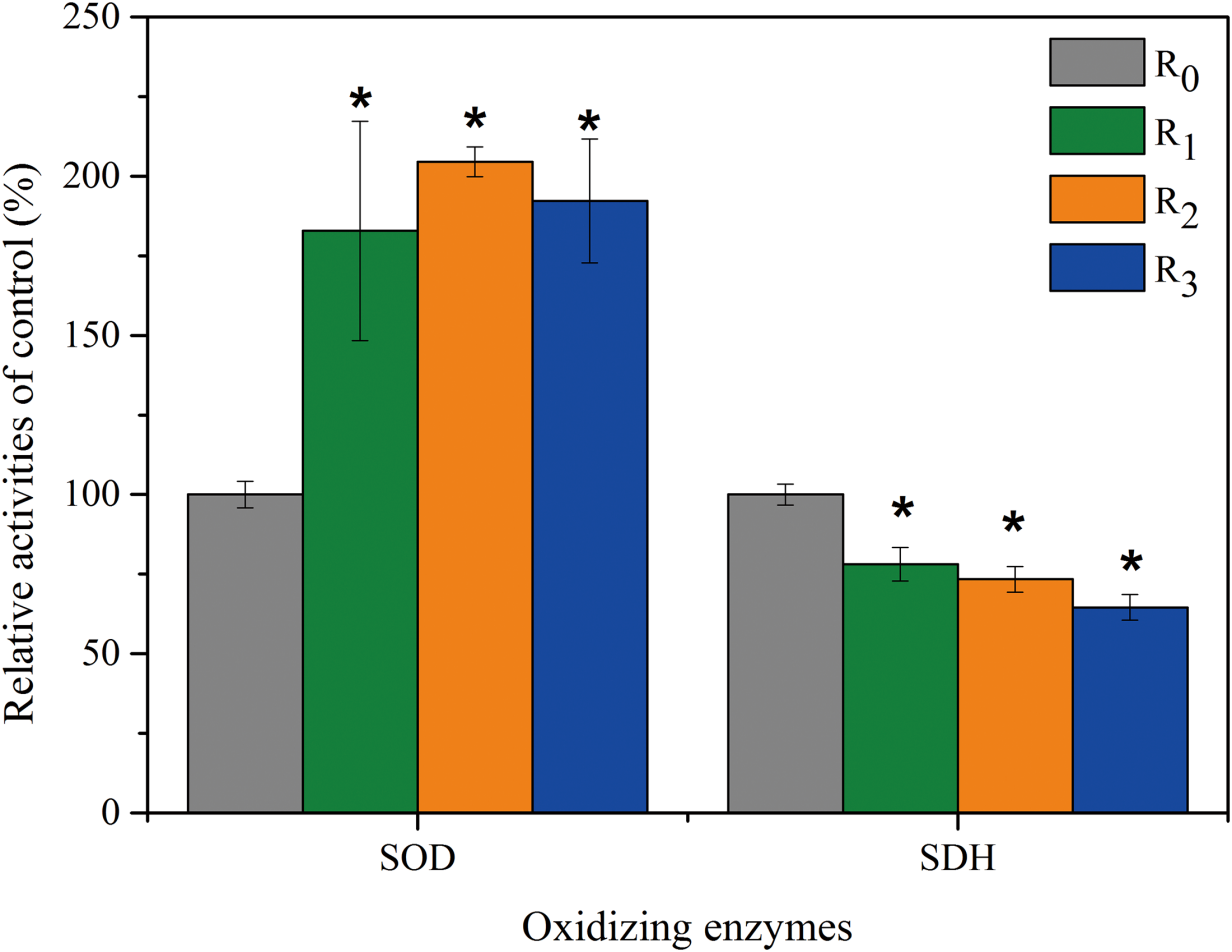
Relative activities of oxidizing enzymes in activated sludge during the stable stage. The enzyme activity of the control is regarded as 100%. The relative activity is presented in the percentage of enzyme activity value in samples with enzyme activity value in the control. The relative activity is over 100% indicating an increase of enzyme activity (compared to the control). The relative activity is less 100% indicating a decrease of enzyme activity (compared to the control). asterisks (*) indicating the statistical difference from the control (*p* < 0.05).

SOD is a key antioxidant enzyme which could indicate the oxidative stress [20]. SDH plays an important role in the oxidation process [34]. SOD and SDH were extracted from activated sludge at the stable stage to assess the oxidative damage to cell membranes. Fig 1 illustrates the changes of SOD and SDH. The activity of SOD (U mg protein^-1^) increased from 25.50±8.87 (R_0_) to 46.62±8.79 (R_1_), 52.15±1.20 (R_2_) and 49.02±4.95 (R_3_), respectively. The results indicated that environmentally relevant concentrations of selected NSAIDs stimulated SOD activity, suggesting that the three NSAIDs could induce the oxidative stress of microorganisms in activated sludge. Oxidative stress response can also be used to indicate low to moderate toxicity of selected pharmaceuticals (especially DCF) by increasing activities of oxidative stress enzymes [20]. SOD can reduce the toxicity associated with radicals by clearing the free oxygen radicals [35]. Zhang et al. [36] reported that SOD may be more sensitive to wastewater toxicity than cell membrane integrity and to cell density when treating municipal WWTPs. Thus, it is possible that microorganisms can reduce the toxicity of selected NSAIDs by increasing SOD activity.

As shown in Fig 1, the activity of SDH (U mg protein^-1^) decreased from 25.25±7.14 (R_0_) to 19.72±1.33 (R_1_), 18.53±1.02 (R_2_) and 16.31±1.00 (R_3_) (Fig 1). SDH existing in many prokaryotic cells is an important enzyme in the tricarboxylic acid cycle. The decrease in SDH activity was possibly attributed to selected NSAID damaged mitochondrial function and to the intermediary metabolism of microorganisms. Similarly, it was found that SDH activity was reduced with the presence of heavy metals, pesticides and other organic pollutants in sludge-supplemented diet-fed animals [37]. The SDH activity had a decreasing tendency from R_0_ to R_3_, which indicated that the mixtures of NSAIDs might cause larger toxicity to microorganisms. This result was supported by Wang and Gunsch [38], who investigated the impact of four pharmaceutically active compounds individually and in mixtures on the performance of SBRs and found that the inhibition of microorganisms was observed only when pharmaceuticals were introduced as the mixtures.

#### Effect of selected NSAIDs on EPS content and live/dead cell distributions

**Fig 2:**
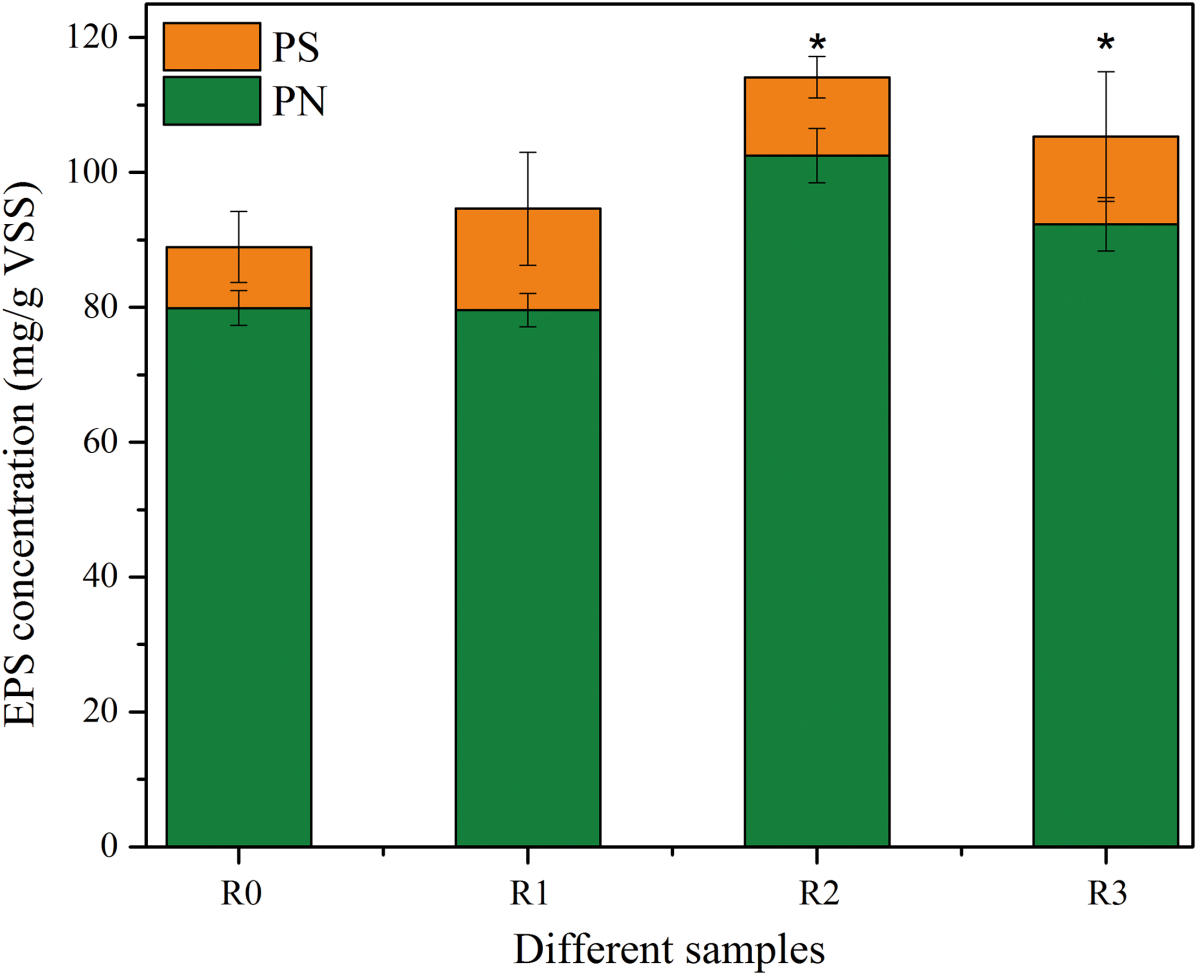
Changes of EPS content in each sludge sample. EPS content is presented by the sum of PN and PS. PN and PS are normalized by volatile solid sludge (VSS). asterisks (*) indicates statistical difference from the control (*p* < 0.05).

It was often accepted that extracellular polymeric substances (EPS) were the important components to evaluate when examining the physicochemical and biological properties of biomass [39]. In this study, EPS content was calculated by the sum of PN and PS [25]. Fig 2 describes the changes of EPS content extracted from activated sludge during the stable stage. It was evident that EPS content improved with the addition of pharmaceuticals compared to the control (88.94 mg g VSS^-1^), which were 94.62, 114.13 and 105.35 mg g VSS^-1^ in R_1_-R_3_, respectively. The increase of EPS content with the combination of selected NSAIDs indicated that the mixtures of selected NSAIDs might improve the toxicity to microorganisms, which could stimulate microorganisms to produce more EPS content. It was found that EPS content increased to form a network structure outside cells protecting cells from the damage of tetracycline or sulfamethoxazole [15]. Delgado et al. [19] reported that the production of EPS content was a reaction to environmental stress, and EPS content was an important part of sludge flocculation. The EPS content inside sludge flocs might be a protective barrier for microorganisms exposed to the selected NSAIDs. It was also found that bacteria produced more EPS content to protect themselves from heavy metal toxicity [40] or antibiotics toxicity [15]. EPS content decreased from R_2_ to R_3_, which indicated that the NSAIDs mixtures might have already damaged the microorganism growths. This finding is also supported by the results of the decreased SDH activity with the addition of selected NSAIDs. Quan et al. [41] also reported that low toxicity may increase the amount of EPS content, but high toxicity may reduce it.

**Fig 3:**
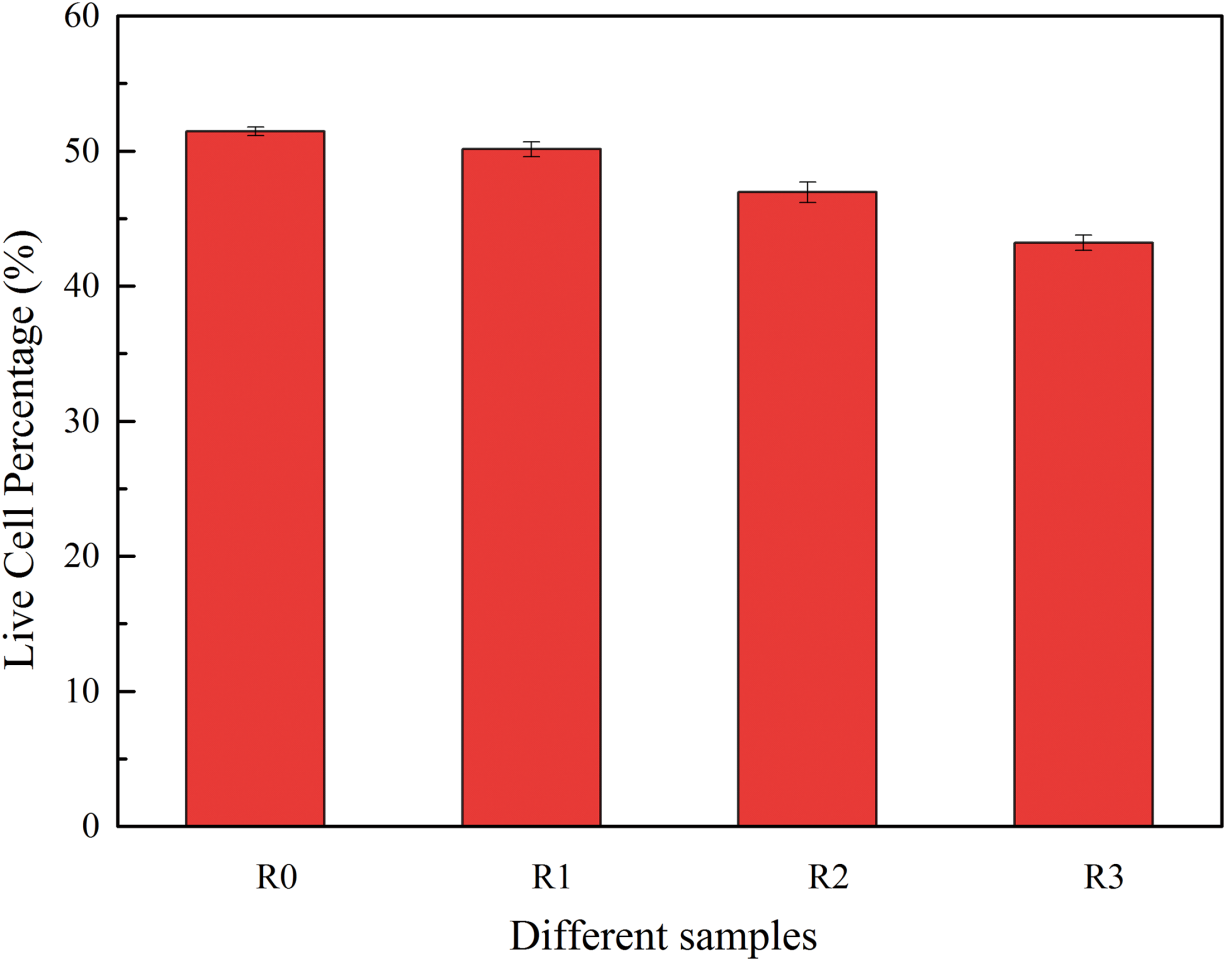
Percentages of live/dead cells in each sludge sample. Twenty random fields were chosen and observed, and the rate of live/dead cells in activated sludge was calculated by quantifying the area of each image. The final results indicate the percentage of live cell.

The ratio of Live/Dead cells in activated sludge was determined at the stable stage. The percentages of live cells in sludge samples are shown in Fig 3. It was seen that percentages of live cells decreased with the addition of selected NSAIDs. The result demonstrated that pharmaceuticals had toxicity in the microbial community and the mixture of pharmaceuticals did more damage to the live cells, which corresponded to the changes of EPS content and the activity of SOD and SDH with the addition of DCF, IBP and NPX. Many studies used Live/Dead cell ratios to determine (at least partially) the toxicity of compounds, membrane integrity and microbial activity [38, 42, 43]. Louvet et al. [42] used live/dead cell distributions to study erythromycin time-kill activity and found that living bacteria decreased from 94% (SD 3.8) to 67% (SD 3.1) with the exposure of 100 μg L^-1^ erythromycin after 45 min. Similarly, there was a decrease of the live/dead cell distribution ranging from 16% to 10% with additions of 1 and 10 uM of ketoprofen, naproxen, carbamazepine and gemfibrozil indicating that four commonly occurring phACs affected membrane integrity [22].

### Microbial community characteristics

#### Effect of pharmaceuticals on bacterial diversity

The Shannon-Wiener index was used to represent bacterial diversity, which was 1.39 (the control), 1.49 (5 μg L^-1^ DCF), 1.53 (5 μg L^-1^ DCF+5 μg L^-1^ IBP) and 1.58 (5 μg L^-1^ DCF+5 μg L^-1^ IBP+5 μg L^-1^ NPX). The result indicated that environmental concentration of NSAIDs could stimulate the bacterial diversity. Zhang et al. [15] observed that the maximum Shannon-Wiener index occurred in SBRs with the addition of 5 μg L^-1^ tetracycline and sulfamethoxazole. Huang et al. [44] used two lab-scale anaerobic-anoxic-oxic systems containing 300 μg L^-1^ of tetracycline to investigate bacterial diversity, showing that microbial diversity increased after tetracycline addition. In fact, with the increase of pharmaceutical concentration to 10 mg L^-1^, the bacterial diversity would be inhibited [15]. Thus, microorganism adaptability plays a role within limits, and a detailed threshold value of pharmaceutical concentration needs further investigation.

#### Effect of selected NSAIDs on microbial community at phylum level

**Fig 4:**
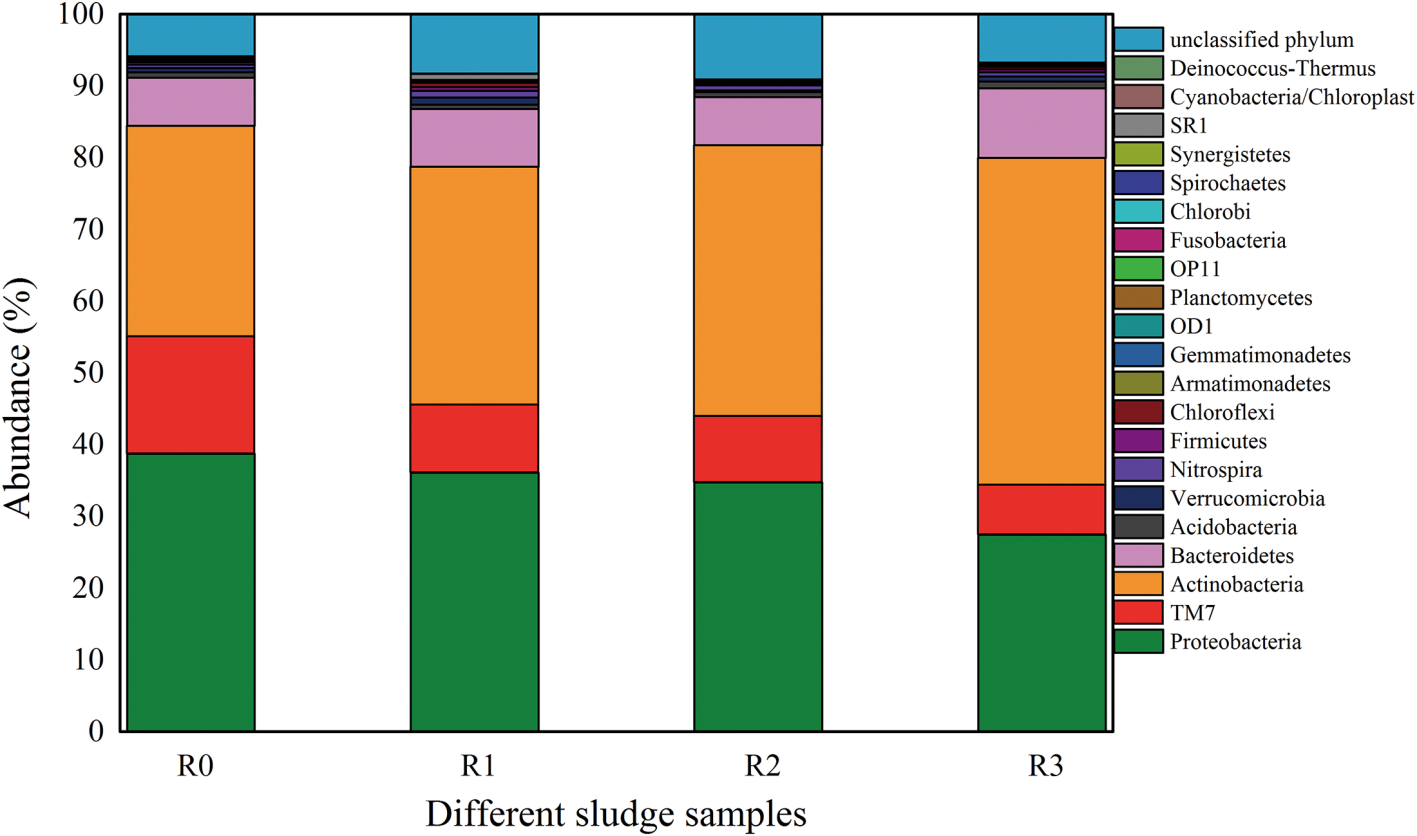
The relative abundances of bacteria in sludge samples of stable stage at phylum level. The abundance is presented in terms of percentage in total effective bacterial sequences in each sample. The color bar indicates the range of the percentage of a phylum. The sum of all phyla and unclassified phylum is 100%.

Microbial community changes in activated sludge at phylum level are illustrated in Fig 4. It is evident that Proteobacteria, Actinobacteria, TM 7 and Bacteroidetes were the four most abundant phyla in all samples, and they are often observed in activated sludge [15, 45]. The abundance of Proteobacteria, Actinobacteria, TM 7 and Bacteroidetes of the control were 38.71%, 29.38%, 16.36% and 6.65%, respectively. Compared to the control, the abundance of Proteobacteria and Bacteroidetes had no distinct change (*p* > 0.05) in R_1_ and R_2_, while the Proteobacteria decreased from 38.71% to 27.46% in R_3_ and the Bacteroidetes increased from 6.65% to 9.71% in R_3_. The Actinobacteria abundance increased from 29.38% in R_0_ to 45.56% in R_3_. TM 7 abundance steadily decreased from R_0_ to R_3_ until it finally reached its lowest percentage of 6.93% in R_3_. The decrease of Proteobacteria indicated that Proteobacteria might not adapt in the presence of selected NSAIDs. Actinobacteria show an increase from R_1_ to R_3_, suggesting that Actinobacteria could adapt well with the presence of selected NSAIDs. Recently, Zhang et al. [46] reported that Actinobacteria abundance increased in IBP-enriched planed beds compared to the control. Pala-Ozkok et al. [47] also reported that the continuous exposure of activated sludge to erythromycin for three days caused preferential growth and predominance of members of Actinobacteria (increased from 48% to 22%). Actinobacteria could hydroxylate DCF to different products referring to the activity of a cytochrome P450 enzyme [48]

Bacteroidetes are heterotrophic microorganisms and could degrade high molecular weight organic compounds like petroleum hydrocarbons [49]. The higher percentage of Bacteroidetes in R_3_ might be explained by the increased concentration of the mixtures of selected NSAIDs. The explanation is supported by [50], who found that Bacteroidetes increased at an urban site with higher concentration of complex organic compounds. The features and functions of TM 7 are limited [51].

Some special but not abundant bacteria (data not shown), such as Nitrospira, Firmicutes and Chloroflexi, were gradually increased, but others, like Armatimonadetes and Gemmatimonadetes, were gradually below the detection limit. The increase of some microorganisms was probably due to their ability to degrade pharmaceuticals. It was found that Nitrospira and Firmicutes served as an important function in the treatment of wastewater with pharmaceuticals [21]. Similarly, Zhang et al. [46] also found that Firmicutes, Actinobacteria and Proteobacteriain had the best adaptation to the changed external environment in wetland systems. Nitrospira, belonging to nitrite oxidizing bacteria (NOB), play a key role in the secondary step of nitrifying [52].

#### Effect of pharmaceuticals on microbial community at genus level

**Fig 5:**
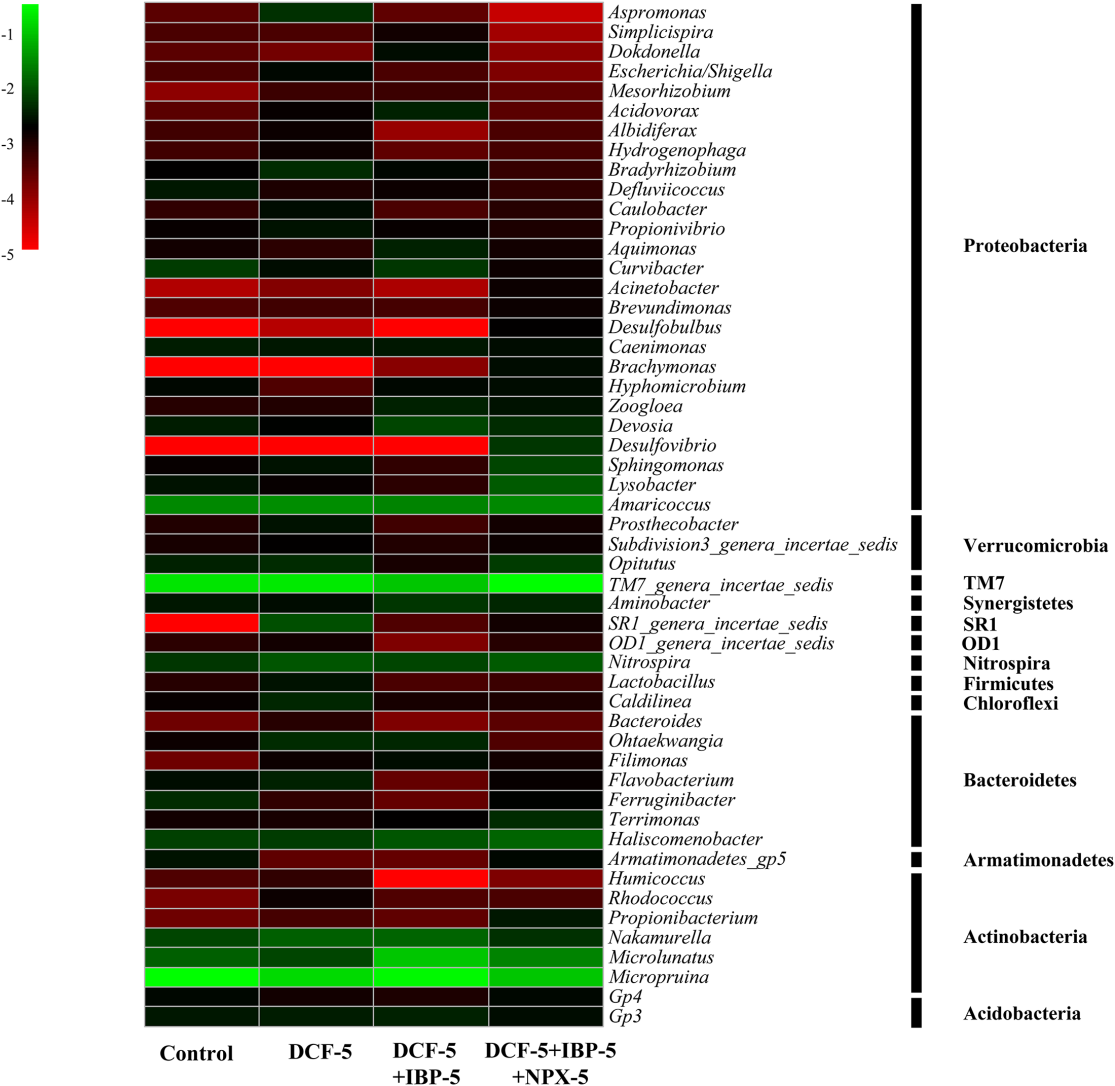
Heat map of genera occurring at >0.2% abundance in at least one sludge sample. The color bar indicates the range of the percentage of a genus in each sample, based on the color key (log 10 scale) at the top right corner. Genera with abundance >0.2% were selected in each sample.

The heat map of bacterial abundance at the genus level is shown in Fig 5. It was observed that *Micropruina* makes up a large part in activated sludge, which kept the decreasing trend from 29.53% to 14.90%, 14.21% and 9.50% in R_0_, R_1_, R_2_ and R_3_, respectively. *Micropruina*, a glycogen-accumulating organism, initially was isolated from an activated sludge reactor, which could indicate the biological phosphorus removal activity [53]. *Nakamurella*, also affiliated with Actinobacteria phylum, decreased from 1.31% (R_0_) to 0.46% (R_3_). Tian et al. [13] reported that *Micropruina* and *Nakamurella* had the ability to accumulate energy-storage chemicals when they were exposed to harsh conditions. *TM7_genera_incertae_sedis*, *Amaricoccus*, *Haliscomenobacter* and *Microlunatus* were the dominant genera in all sludge samples, which increased with the addition of pharmaceuticals. *Amaricoccus* and *Haliscomenobacter* were reported capable of utilizing sulfamethoxazole as a secondary carbon source in combination with acetate [54]. *TM7_genera_incertae_sedis* increased from 16.36% (R_0_) to 27.38% (R_3_), which indicated that the addition of pharmaceuticals could stimulate the abundance of *TM7_genera_incertae_sedis*. However, the contribution of *TM7_genera_incertae_sedis*on in the aerobic decontamination of wastewater was not clear [12]. In this study, the abundance of *Zoogloea*, affiliated with Proteobacteria phylum, had an augmentation under the pressure of pharmaceuticals, increasing from 0.08% (R_0_) to 0.33% (R_3_). It was known that *Zoogloea* can block toxic contaminants using their exocellular matrix [9]. Wang et al. [31] indicated that *Zoogloea* was considered as a functional bacterium in a granular sludge membrane bioreactor with the addition of pharmaceuticals, which could degrade antibacterial and anti-inflammatory organic matter. The abundance of *Desulfovibrio* and *Desulfobulbus* were 0.01% in the control (R_0_), increasing to 0.53% and 0.17% in R_3_, which belong to sulfate-reducing bacteria [55].

The above observations indicated that selected NSAIDs affected not only microbial activities but also microbial community. Actinobacteria and EPS increased with the addition of selected NSAIDs. It might be concluded that Actinobacteria might play an important role in producing EPS. Zhu et al. [25] observed that the abundance of Actinobacteria and EPS content increased in the biofilm sloughing and updating stage and indicated that the increased Actinobacteria and EPS were conducive to resist environmental stress. Cycoń et al. [56] speculated that the low activity of dehydrogenase with the presence of NSAIDs might be caused by the death of the drug-sensitive part of the microbial population. Analogically, the decrease of SDH activity might be associated with the decreased Live/Dead cell rate with the addition of NSAIDs. Due to the complexity of microorganisms, the results of microbial changes always are comprehensive. Moreover, further investigations are needed to expound the impact of NSAIDs on microbial community and activity.

## Conclusions

With the presence of selected environmental concentrations of NSAIDs in SBRs for 130 d, wastewater treatment efficiencies were not affected except for the reduction of TN removal. The results of the ratio of Live/Dead cell and oxidizing enzymes activities provided that environmental concentrations of selected NSAIDs caused toxicity to microbial communities, and the mixtures of three NSAIDs improved the toxicity. EPS content was increased to protect microorganisms from selected NSAID toxicity. The Shannon-Wiener diversity index increased with the presence of selected NSAIDs, which indicated that the environmental concentration of NSAIDs stimulated microbial diversity. Proteobacteria, Actinobacteria, TM 7 and Bacteroidetes were the dominant species in the activated sludge. Actinobacteria and Bacteroidetes were enriched with the presence of selected NSAIDs.
